# Reduction in Step Height Variation and Correcting Contrast Inversion in Dynamic AFM of WS_2_ Monolayers

**DOI:** 10.1038/s41598-017-18077-4

**Published:** 2017-12-19

**Authors:** Kyle Godin, Christian Cupo, Eui-Hyeok Yang

**Affiliations:** Department of Mechanical Engineering, Stevens Institute of Technology, Hoboken, New Jersey 07030 United States

## Abstract

A model has been developed to account for and prevent the anomalies encountered in topographic images of transition metal dichalcogenide monolayers using dynamic atomic force microscopy (dAFM). The height of WS_2_ monolayers measured using dAFM appeared to be increased or decreased, resulting from the interactions between the tip and the surface. The hydrophilic SiO_2_ substrate appeared higher than the weakly hydrophilic WS_2_ when the tip amplitude was low or at a high set point (high force). Large amplitudes and low set points corrected the step height inversion, but did not recover the true step height. Removing water from the sample resulted in an order of magnitude reduced variation in step height, but the WS_2_ appeared inverted except at low amplitudes and high set points. Our model explains the varying step heights in dAFM of TMDs as a result of varying tip-sample interactions between the sample and substrate, in the presence or absence of capillaries. To eliminate contrast inversion, high amplitudes can be used to reduce the effect of capillary forces. However, when capillaries are not present, low amplitudes and high set points produce images with proper contrast due to tool operation in the repulsive regime on both materials.

## Introduction

Transition metal dichalcogenides (TMDs) are a class of layered materials from which a single layer can be isolated with a thickness less than 1 nm. TMD monolayers have unique optical and electrical properties including high absorption relative to their thickness^1^, direct band gaps^2–4^, rapid charge separation^5^, and can even be ferromagnetic^6–8^ or superconducting^9–11^. Due to their thinness, monolayer TMDs interact strongly with their environment; this strong interaction makes characterization of trapped contaminants, surface adsorbed contaminants, water layers, and the TMD-substrate step height necessary to predict and understand device operation^12–16^. Dynamic atomic force microscopy (dAFM), in which a nanoscopic tip is driven sinusoidally, can measure topography while providing a plethora of additional data channels for characterizing the surface absorbed contaminants. dAFM allows for simultaneous measurement of surface potential^17^, long range attractive forces^18^, dispersive and conservative forces^19^, and performing magnetic^20^ and electrical measurements^17,18^. Contact AFM can provide a subset of these measurements, but cannot be used to characterize tip-sample forces in the region of attractive force above the surface where the tip snaps into contact, within 1–3 nm of the sample surface^21^.

In topographic measurements of TMDs on substrates, the step height from the substrate to the top of the TMD is often assumed to be the same as the bulk interlayer spacing, which can be measured accurately by X-ray diffraction. However, due to possible contaminant layers, incomplete bonding to the substrate, and different forces between the TMD and substrate, the measured step height is not necessarily the same as the bulk interlayer separation. Variation in AFM step heights of TMDs have not been systematically investigated to date; reports in literature include a wide range of measured WS_2_ thicknesses, 0.6 nm – 0.92 nm^22,23^, as opposed to the bulk interlayer spacing of 0.67 nm. Even with a perfectly conformal and contaminant-free TMD monolayer on a substrate, the measured thickness can vary and the WS_2_ can appear lower than the substrate (*i.e*., an inverted image), a phenomenon known as contrast inversion^24–26^. This is contrary to measuring a monolayer step within the same material, where step height anomalies are not observed^27,28^, indicating that varying step heights are an artifact of stepping from one material to another. Thin film and nanoparticle topographies from many sample-substrate systems have shown similarly varying height and contrast inversion, including block copolymers, nanoparticles, and self-assembled monolayers on Au and mica^24–26,29–33^. For these other material systems, the measured layer thicknesses and nanoparticle heights have been found to be dependent on tip amplitude and force^34^. In graphene on SiO_2_, varying step heights have been attributed to capillary effects^35^ or to compressed trapped contaminant layers between the graphene and the substrate^36^. These trapped water layers have been directly observed^37,38^. It was reported that the effect of capillary forces was minimized by operating AFM in the repulsive regime on both sample and substrate^35^. The trapped contaminant layers were shown to be compressed to the substrate by the tip force^36^, which explains why high tip forces produced results closer to the bulk interlayer spacing. Step height, thickness, and topography measurements in AFM of TMD monolayers exhibit many artifacts and features which have not been systematically studied.

Here we report our systematic study on dAFM topography of monolayer TMDs. We show that step height errors are caused primarily by varying surface water layer thicknesses on the less hydrophilic TMD and hydrophilic SiO_2_. We investigate the effect of tip amplitude and set point on measured step heights of TMDs on SiO_2_. We use amplitude-phase-distance (APD) data to produce force distance curves which allows for a quantification of the attractive force experienced by the tip when approaching the surface. We demonstrate that the variation in step heights is reduced by an order of magnitude after partial removal of surface water, which decreases the capillary forces experienced by the tip. Finally, we define the tip-sample interactions which result in contrast inversion and prescribe operational settings in terms of appropriate amplitudes and set points which restore proper contrast.

## Results

**Figure 1:**
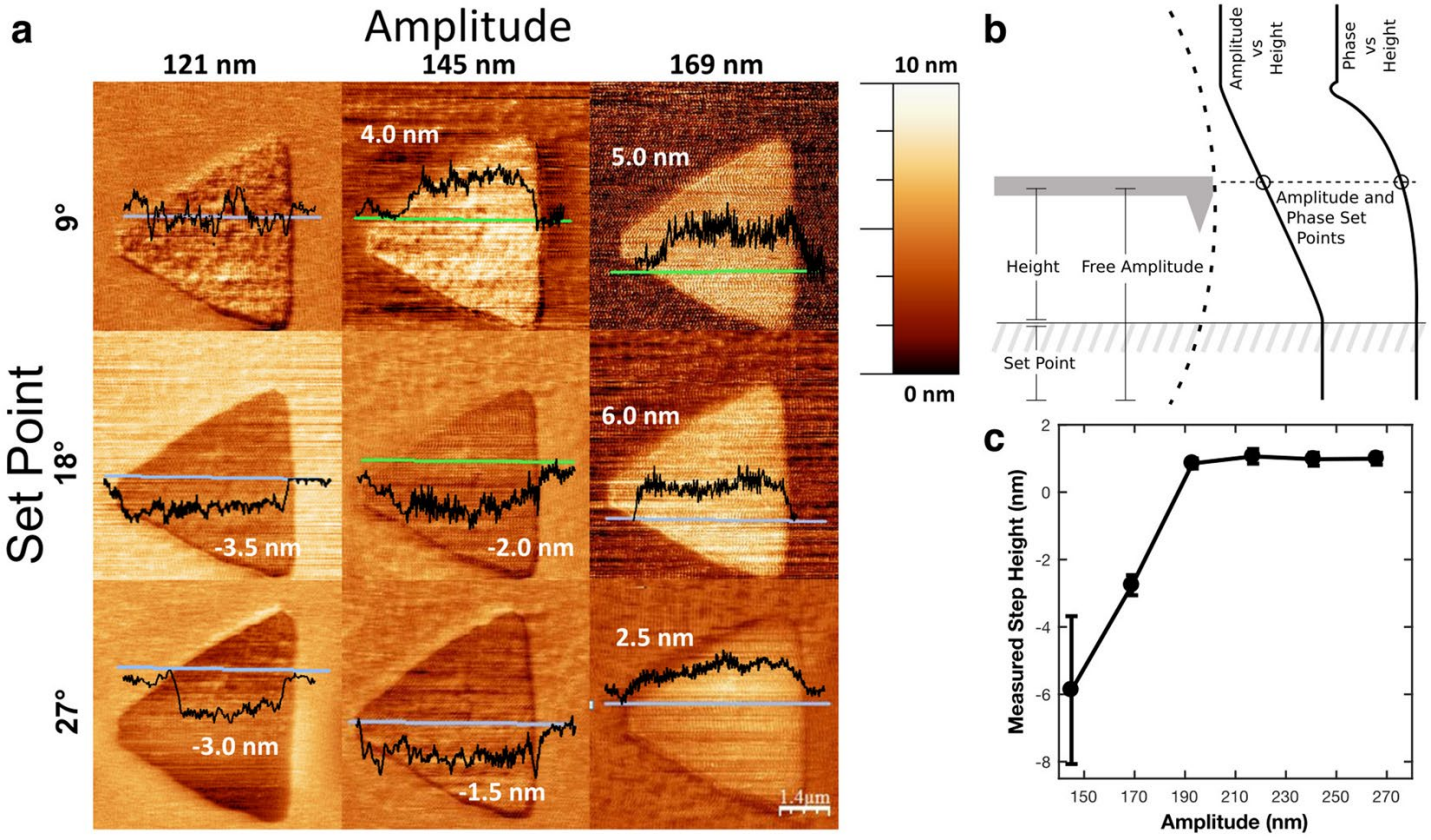
Observed step heights for single crystalline WS_2_ monolayers grown on SiO_2_ using phase feedback based on set point and tip amplitude. (**a**) AFM topography images of a single crystalline WS_2_ monolayer triangle grown on SiO_2_ with step heights ranging from +6.0 nm to −3.5 nm (with WS_2_ appearing below the substrate). (**b**) Definitions for height, amplitude, phase set point, and amplitude set point. The left side illustrates the AFM tip, with the height being the tip-sample distance (piezo height) and the set point as the amplitude reduction. The right side illustrates how the amplitude and set point change with height, with a defined amplitude or phase used as the set point, corresponding to a specific approach distance (**c**) Step height vs. tip amplitude for phase feedback with a constant set point of 9°. The step height transitioned from negative to positive when increasing the tip amplitude.

Figure 1a exhibits the range of observed step heights for single crystalline WS_2_ monolayers grown on SiO_2_ (CVD WS_2_) in phase feedback, based on set point and tip amplitude, the two essential parameters for dAFM. In dAFM operation, as the tip begins to interact with the sample, the tip amplitude is reduced, the resonant frequency can shift, and the phase can increase or decrease from its in-air value. Any of those three variables can be used for feedback when scanning. While the amplitude is not sensitive to the sign of the tip-sample force, the phase will increase with a net positive tip-sample force and the phase will decrease with a net negative tip-sample force^29^. We chose phase feedback to ensure operation in the repulsive regime. We have defined the oscillation amplitude as the distance the tip travels from the lowest to highest point (Fig. 1b). We have defined the set point as the percent reduction in amplitude when using amplitude for feedback and the angle reduction from 90° when using phase feedback (Fig. 1b). For both feedback modes, high set point means high force. The height is the piezo height which corresponds to the tip-sample distance plus an arbitrary offset. As shown in Fig. 1a, the set point and amplitude can cause an increase or decrease in the apparent thickness, in agreement with other reports^34,39^. In this work, the AFM images were taken consecutively on the same sample (CVD WS_2_), with tip amplitudes 121 nm, 145 nm, and 169 nm and the set point at 9°, 18°, and 27° (phase feedback mode; amplitude feedback produced a similar range of results). The tips used were pulled SiO_2_ fibers with high Q (~1600) and the range of amplitudes used produced results comparable to a range of 20–60 nm for common Si tips. AFM images were corrected for line-by-line offset and parabolic error from the piezo scanner using the “flatten” function of WSxM^40^. Four of the nine images had negative step heights, *i.e*., the TMD was imaged as if it was pressed into the substrate below the surface. As can be seen, changing either the set point or the amplitude affected the step height measured on the same sample. We cannot attribute the step height variation or contrast inversion to sample roughness, as the SiO_2_ substrate roughness was < 0.5 nm RMS and the WS_2_ roughness was <0.5 nm RMS, excluding bilayer defect regions. For the same experiment with exfoliated materials, large steps (>6 nm) were recorded and no contrast inversion was observed over a wide range of set points and amplitudes which we attribute to contaminants trapped between the TMD and the substrate. High vacuum annealing reduced the observed height of exfoliated samples and caused them to appear inverted under certain amplitudes and set points. We have also worked with graphene but have never observed inversion, only obtaining measured heights greater than the bulk interlayer spacing of 0.34 nm. We also used contact mode AFM, which is less affected by water layers^26^ and does not produce contrast inversion^36^. However, in our experiments it also did not result in a consistent, unambiguous measurement for the step height.

Figure 1c examines the transition of the contrast from a negative to positive step height. We generated phase feedback dAFM topographic images of a non-annealed CVD WS_2_ monolayer at varying amplitudes from 145 nm to 266 nm in intervals of 24 nm (Fig. 1c). A constant set point of 9° was used for all images, chosen as a mid-range value. For each image, twenty step heights were obtained and averaged for the measured step height, while error bars were calculated as the standard deviation. As shown in Fig. 1c, the measured step height of the WS_2_ was inverted at low tip amplitudes and transitioned to correct contrast at a critical amplitude. Figure 1a shows that this critical amplitude depends on the set point. At the same time, the variability in the measured step height was larger when scanning at the low amplitude of approximately 150 nm.

To reduce the effect of a water layer, we annealed a sample in air at 130 °C for 30 minutes. After annealing, when scanning with a wide range of typical amplitudes (60–362 nm) and set points (9–23°), contrast inversion was observed for all combination of parameters. The correct contrast was restored using low amplitudes (<20 nm) and high set points (>30°), directly contrary to the case before annealing, which indicates that the surface forces between the tip and sample are different before and after annealing. This is summarized in Figure S1, which repeats the experiment in Fig. 1a but for an annealed sample. After annealing, it is shown that low amplitudes exhibit correct contrast. Placing the samples in a low humidity environment did not effectively remove surface water^41^. In addition, the measured (apparent) step heights for the annealed samples had a smaller variation (<0.5 nm standard deviation), centered higher than the interlayer spacing value of 0.67 nm. We investigated the remaining variation through amplitude-phase-distance (APD) curves.

Theoretically, the APD curves can be used to compensate for step height measurement errors^24,26,42^. This is accomplished by finding the height difference between points of equal phase on the two different materials. We found that, even with annealing, the variation in single point APD curves taken on different parts of a sample was on the same order as the step height (0.67 nm). Without annealing, the variation was an order of magnitude higher, 3–6 nm. Thus, single point APD curves cannot be used in this case to compensate for the differences in tip-sample forces between the WS_2_ on SiO_2_ and bare SiO_2_. We expect that a pristine WS_2_ on SiO_2_ sample could have step heights corrected through APD curves, as in other material systems which had tip-sample forces dominated by van der Waals forces^24,26,42^.

**Figure 2:**
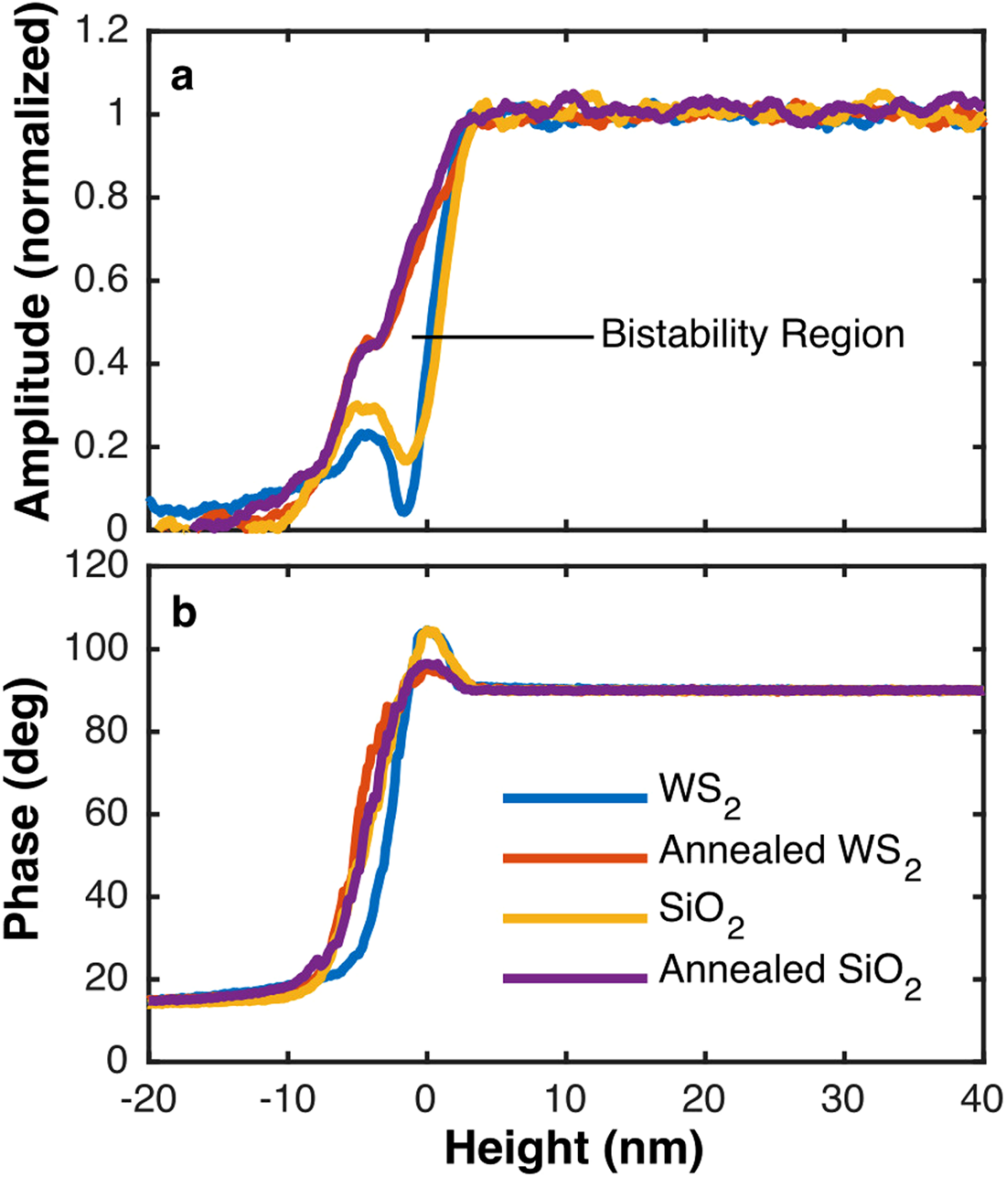
Normalized Amplitude vs Height and Phase vs. Height for SiO_2_ and WS_2_, annealed and non-annealed. The height is the piezo height, shifted to be zero at the peak phase, to compensate for the arbitrary piezo height offset. The non-annealed samples exhibit strong attractive forces (phase >90°) and severe amplitude reduction in the attractive region, implying stronger attractive forces than in annealed samples. The annealed samples exhibit weaker attractive forces and only partial amplitude reduction in the attractive region.

To investigate the effect of the surface water layer on tip-sample interactions, the APD curves on both WS_2_ and SiO_2_ were compared before and after annealing the samples in air at 130 °C for 30 minutes (Fig. 2). The effect of water adsorbed on the surface can be clearly seen in the APD curves. The WS_2_ samples were CVD WS_2_ monolayer triangles of side length 5–50 nm on SiO_2_ that had been stored in an Ar environment. We used dynamic APD curves to enable investigation of the tip-sample forces near the sample surface which are inaccessible to the static mode due to tip snap-in. The phase, amplitude, and piezo height were recorded as the tip approached the sample from a non-interacting region (*e.g*., over 100 nm from the surface). It should be noted that the variations were not due to tip damage; tip damage was minimized by minimizing the approach distance. In these measurements, the retract curves were not used as the tip-sample forces were obscured by additional effects^31^. The experiment was repeated several times on different days with different WS_2_ samples. A key observation was that lower tip amplitudes resulted in larger tip-sample interactions in the attractive region (phase >90°) on the APD curves. The data shown are with tip amplitudes ~13 nm, a relatively low value and near the minimum that the tool could be run due to noise in the piezoelectric oscillation sensors. The tip resonance frequency was observed to shift by ~1 Hz from the frequency with free amplitude while in contact. If there was a large shift in the resonance frequency from the original locked in value, the results would be invalid as the amplitude reduction and phase shift would be a mixed effect from tip-sample forces and a frequency shift. Force distance curves and the dissipation coefficient were interpreted from the method of Payam *et al*.^43^. The data plotted against piezo height are shown in Fig. 2, with the piezo height shifted to make H = 0 the peak of the phase in the attractive region. The true sample surface is necessarily between the onset of the attractive region, on approach, and the transition to the repulsive regime. The peak of phase is taken as an approximation of the location of the sample surface to allow curves to be compared properly. Additionally, the phase was shifted to be 90° in air, as expected from a damped driven spring in resonance. The raw phase was measured by convolving the driving phase with the tip phase and therefore was 0° in air.

When starting to interact with the sample, the phase initially increased and the amplitude decreased; phase >90° means that the net tip-sample interaction is attractive and phase <90° means the net interaction is repulsive^44^. While the phase is sensitive to the sign of the tip-sample forces, the amplitude is always reduced, a fundamental property which arises in damped driven spring systems^29^. In Fig. 2, differences between the samples with and without annealing are apparent. Annealing reduced the amount of the phase increase in the attractive region by approximately 3x, which implies weaker attractive tip-sample forces in annealed samples. Without annealing the sample, the amplitude reduced rapidly and almost the entire amplitude reduction occurred in the attractive region. With annealing, however, the phase increase was smaller and the amplitude reduction was slower, implying smaller attractive forces. In the annealed sample, the amplitude showed a horizontal region (bistability) at the transition from the attractive to the repulsive regime, as is commonly observed^35,44,45^. After the bistability, the amplitude had a further reduction. We attribute this observation to the existence of water layers on the WS_2_ and SiO_2_ surfaces; this resulted in an attractive force due to surface tension which dominated the tip-sample interactions. Pure van der Waals forces would result in a phase increase equal to or less than observed in the annealed samples. On the annealed sample, the attractive region was wider than on the non-annealed sample. When comparing the width and structure of the attractive region on many trials, it was clear that the phase curve in the attractive region was defined by a short range interaction (<3 nm) and a long range interaction, extending from 1 nm to 6 nm from H = 0. On non-annealed samples, some appeared with only a short range interaction which was about 3 times higher than any phase shift on annealed samples, and some had only the long range component which peaked at up to 70% the height of the short range component. We attribute this to capillaries forming at two length scales, depending on local sample conditions for capillary attachment. On annealed samples, only the long range component appeared and was broadened, extending from 1 nm to 8 nm from H = 0. This implies that the stronger, shorter range forces (attributed to capillaries) were mediated by surface contaminants which were removed through annealing. The broadened attractive region on annealed samples was attributed to a net attractive force when initially in contact, not to longer range forces. In the retract curves (not shown), there was no attractive region on annealed samples and a strong attractive region on non-annealed samples, evidence of effective water removal through annealing. Retract curves were not analyzed closely as they are affected by adhesion, piezo hysteresis, and other effects.

## Discussion

When taking topographical images (2D AFM images) of WS_2_ on SiO_2_, several channels were recorded simultaneously including the amplitude, phase, and height. In the 2D image taken with phase feedback, we observed the amplitude was reduced on WS_2_ compared to SiO_2_ alone at any set point. Using phase feedback ensured that we were in the repulsive regime as the phase is sensitive to the sign of the force. In the absence of dissipative forces, the amplitude should be linear with the sine of the phase^46,47^, but Fig. 2 shows that in our samples the amplitude -sin (phase) relationship was not first order and varied between samples. When recording using phase for feedback, the phase should be held at zero through the entire map, though it can deviate at steps due to a time lag from the feedback loop. Considering the shape of APH curves, which are constrained to decay over a specific height range equal to the free amplitude, the consistently lower amplitude on WS_2_ could be due to either a wider attractive region on WS_2_ or a greater amplitude reduction in the attractive region on WS_2_; we observed the latter in both the annealed and non-annealed cases. If this trend of amplitude reduction were in the contact region it could be attributed to the softness of WS_2_ or to compressing the layer, but instead it occurred in the attractive region. Thus the amplitude reduction on WS_2_ implies a stronger attraction between the tip and WS_2_ than SiO_2_, whether annealed or not.

**Figure 3:**
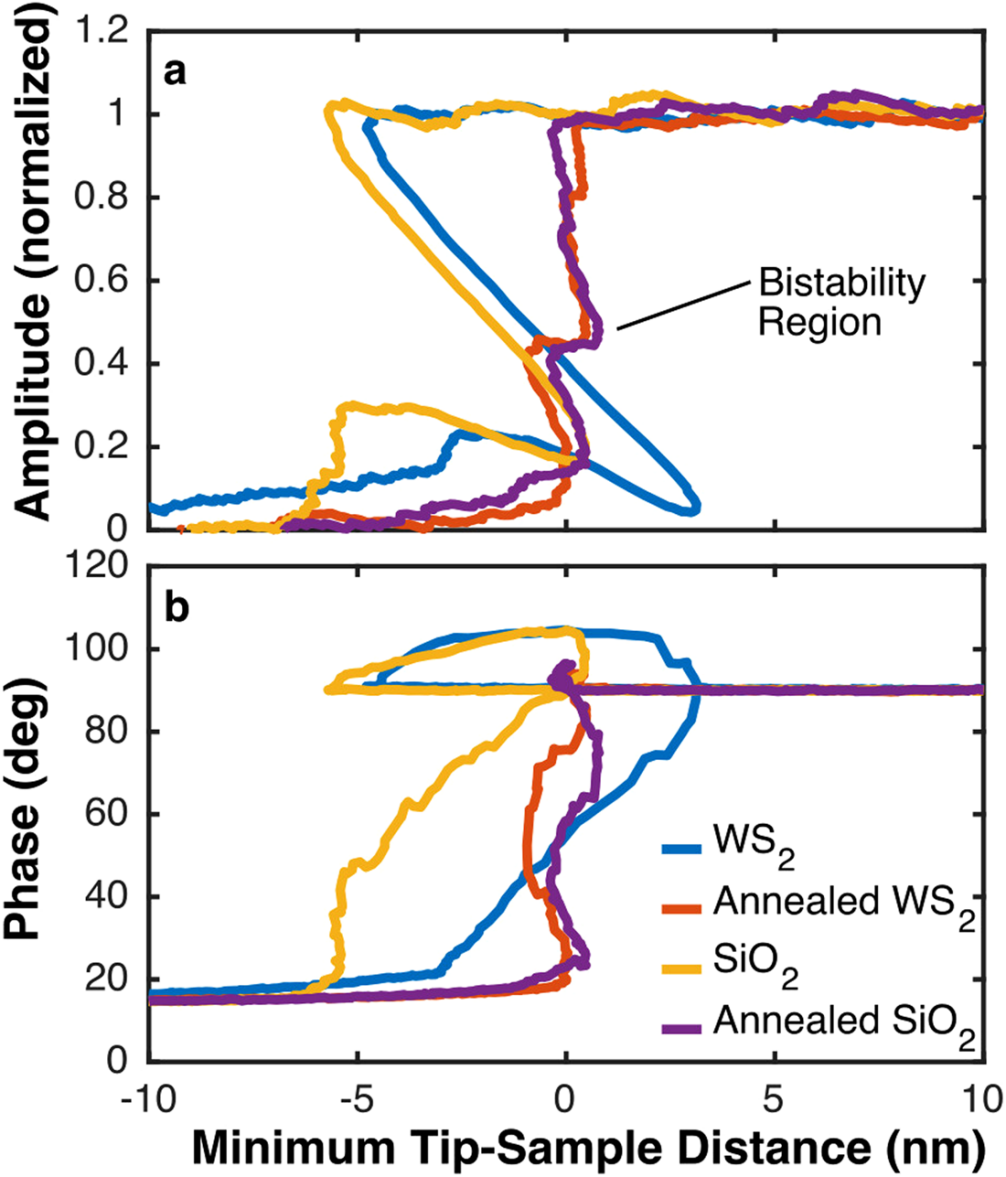
Normalized amplitude and phase vs minimum tip-sample distance. When converting from height to tip-sample distance, the dramatic amplitude reduction in the attractive region for non-annealed samples caused D to be not monotonic. The apparent greater width of the attractive region for WS_2_ is due to more rapid amplitude reduction in the attractive regime.

To produce FD curves, the height was converted to the tip-sample distance, D. In this case, the distance is defined as the minimum distance between the tip and the sample, D = H-A + offset, where H is the height, A is the amplitude, and the offset is to make D = 0 at the sample surface. In practice, D = 0 is set to the location of the peak of the phase in the attractive region. When plotted against D, there was a clear difference between samples from annealed and non-annealed samples (Fig. 3). When in full contact and without long range interactions, the amplitude should reduce concomitantly with the height (dA/dH = 1). In that case, the amplitude and phase curves should be a vertical line at D = 0. In the case of weak long range interactions, such as van der Waals forces, the slope of the amplitude reduction (dA/dH) near to but smaller or greater than 1, which produces nearly vertical but oscillating curves as shown in Fig. 3 for the annealed cases, both WS_2_ on SiO_2_ and nearby bare SiO_2_. For non-annealed samples, we observed dramatic amplitude reduction (dA/dH > 1), which results in an inverted, non-monotonic curve when amplitude and phase are plotted against D, and as a loop on the PD graph (non-annealed samples in Fig. 3). When D is not monotonic, the FD curves cannot be produced from the theory^43^. For non-annealed samples, the data goes to negative D before the attractive region starts, since D = 0 was set to be the peak of the attractive region, which means that FD curves could not be produced for non-annealed samples for the entire region of tip-sample interaction. For WS_2_, the attractive region appeared wider when plotted against D, an artifact of more rapid amplitude reduction (higher dA/dH).

**Figure 4:**
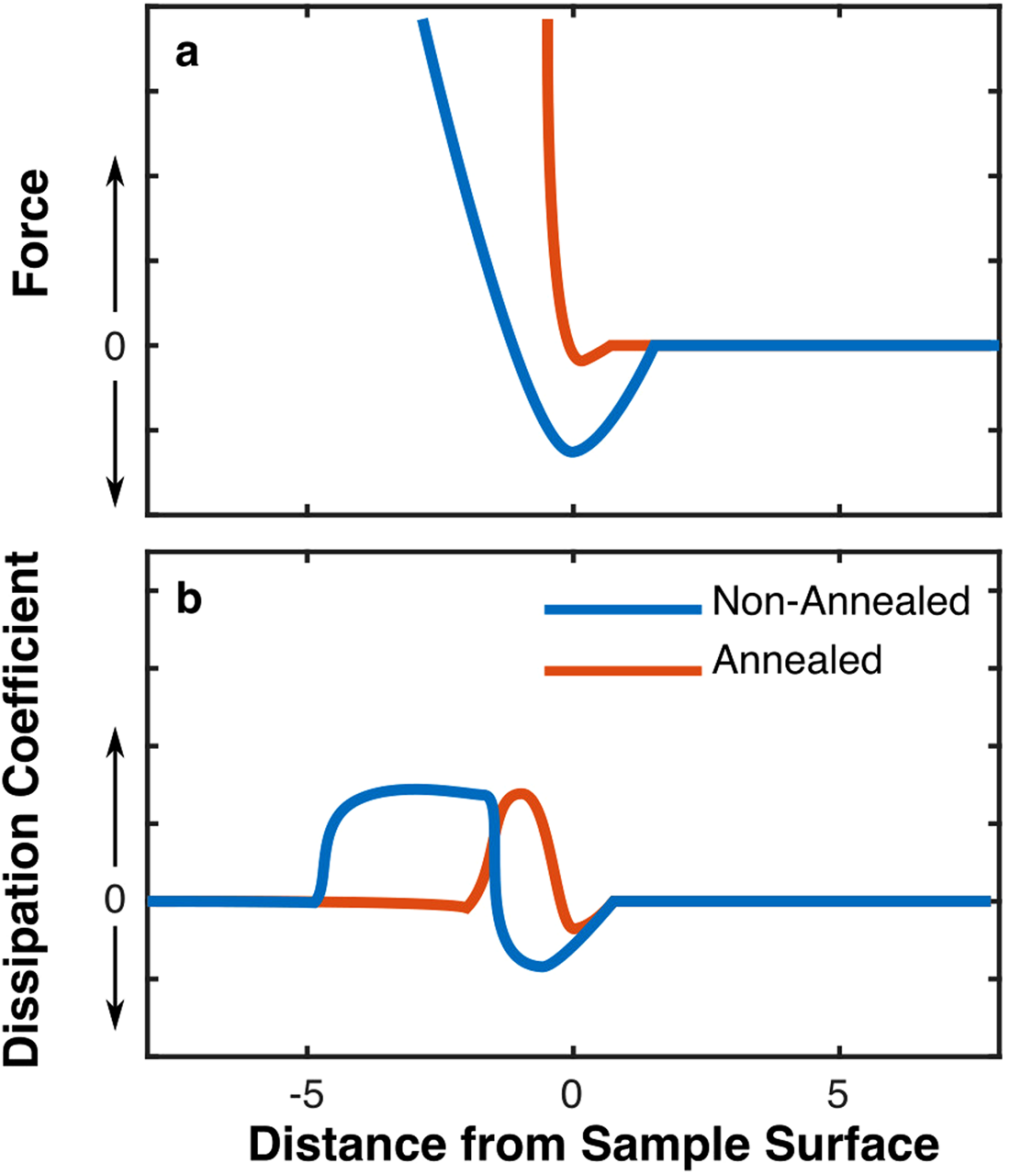
Drawn force and dissipation constant, illustrating major features based on the model in ref.^43^, plotted against minimum tip-sample distance, D. When near the surface the tip is attracted to the sample and is given energy; when well in contact the tip is repelled and energy is dissipated. The non-annealed samples should exhibit a wide region of negative dissipation due to the amplitude reduction in the attractive regime, dA/dH >1.

Figure 4 illustrates the main features of the expected force-distance (FD) curves on top and the non-conservative dissipation factor on bottom, constructed by referring to the model in Payam *et al*.^43^. The model converts dynamic APD curves to an FD curve and dissipation curve. The inverted slope on the APD curves means that the FD curves cannot be calculated from the integral equations, for the data presented in this paper. This is because the equation for the FD curve integrates the APD curves from *d* to infinity, which is not well defined when *d* is not monotonic. In the model, there are three terms in both the models for the force and for dissipation:

1 $$F(d)=2k{\int }_{d}^{\infty }Xdx+2k{\int }_{d}^{\infty }\frac{\alpha \sqrt{A}}{\sqrt{\pi (x-d)}}Xdx-2k\frac{\partial }{\partial d}{\int }_{d}^{\infty }\frac{{A}^{3/2}}{\sqrt{2(x-d)}}Xdx$$

2 $$X=\frac{{A}_{0}}{2QA}\,\cos (\phi )-\frac{1}{2}\frac{{\omega }_{0}^{2}-{\omega }^{2}}{{\omega }_{0}^{2}}$$

3 $${\rm{\Lambda }}(d)=2k\frac{\partial }{\partial d}{\int }_{d}^{\infty }Ydx+2k\frac{\partial }{\partial d}{\int }_{d}^{\infty }\frac{\alpha \sqrt{A}}{\sqrt{\pi (x-d)}}Ydx-2k\frac{{\partial }^{2}}{\partial {d}^{2}}{\int }_{d}^{\infty }\frac{{A}^{3/2}}{\sqrt{2(x-d)}}Ydx$$

4 $$Y=\frac{{A}_{0}}{2QA\omega }\,\sin (\phi )-\frac{1}{2Q{\omega }_{0}}$$

where *d* is the minimum tip-sample distance, *k* is the spring constant, *x* is the ordinate in the amplitude (*A*) and phase (*φ*) data, *Q* is the tip quality factor, and *ω* and *ω*_0_ are the driving and tip resonant frequencies. *α* is a factor approximately equal to 1/8. By analysis of the terms, for both equations 1 and 3, the second term is smaller than the third term by a factor of $\frac{2}{\pi }\frac{1}{8{A}_{0}}\approx \frac{1}{10A}$. With amplitudes of at least 8 nm in all data taken, the second term can be ignored with a maximum of ~1% error introduced. As derivatives and integrals produce terms of the same order because dA/dH ≈ 1, to within a factor of 5, the ratio of Term 3 to Term 1 can be estimated as proportional to *A*_0_. This was observed in data which was integrable (not shown) – Term 1 dominated for free amplitudes less than about 20 nm, and Term 3 dominated for amplitudes larger than that, with a rapid transition between the two. Thus, in the data presented with an amplitude of 13 nm, Term 1 should dominate and the following discussion applies to Term 1. Another difference between Term 1 and Term 3 is that Term 3 is necessarily local due to the factor $1/\sqrt{2(x-d)}$ in the integral which picks out terms where *x* ≈ *d*, while Term 1 depends on the entire data from *d* to infinity.

To evaluate the shape of the FD curve, we first assume that the tip is driven on resonance which means X = 0 far from the sample surface. The sign of X depends on the phase only, with *φ* > 90 corresponding to cos(*φ*) < 0 and thus X < 0. In the attractive region, with *φ* > 90, the FD curve will show negative values, an attractive force. When *φ* < 90, then X > 0, and as the phase reduces to zero the term X goes to infinity. Term 1 depends on the integral of X from *d* to infinity, which means the resulting curve should have an initial attractive region which is quickly dominated by the repulsive force as the phase drops to zero in the repulsive region. The result of applying this analysis to the APD data in Fig. 3 is shown in Fig. 4a (top). In non-annealed samples, the attractive region is narrower, centered closer to D = 0, and the repulsive regime should quickly dominate. This is contrasted to the non-annealed samples which exhibit wide attractive regimes where most of the amplitude reduction occurs, producing much more negative values of X over a wide range of D values, which when integrated produce a wide and deep region of attractive force which is not as rapidly dominated in the repulsive regime, resulting in a smaller slope of the FD curve in that region. Based on the height and width of the attractive regimes, the strength of the attraction was comparable on both materials. If it were possible to construct FD curves from the non-annealed data, they would exhibit approximately 3 times stronger attractive forces since the phase change was 3 times higher in the attractive regime on non-annealed samples.

An assumed and drawn non-conservative energy dissipation factor, similar to the coefficient of *dz/dt* in a damped-driven spring model, is plotted against tip sample distance in Fig. 4b. To evaluate the shape of the dissipation coefficient curve, we again assume the tip is driven on resonance, which means Y = 0 far from the sample surface (where *φ* = 90 and A = A_0_). An interesting case to consider is when A/A_0_ = sin(*φ*) as the tip approaches, the condition for zero dissipation^46^. This agrees with the model, as Y = 0 for all d under that condition. Unlike the FD curve, where the sign depends only on *φ*, the sign of the dissipation depends on the relative values of sin(*φ*) and A/A_0_. If sin(*φ*) > A/A0, then Y is positive. Considering Term 1, Y is integrated from *d* to infinity which, if Y is consistently positive, produces a monotonically decreasing function. The derivative would be negative. Thus, the condition for negative dissipation is a rapid decrease in amplitude, so that sin(*φ*) > A/A0. This is observed throughout the entire attractive region for non-annealed samples, for both WS_2_ and SiO_2_. This is also observed at the beginning of tip-sample interaction for annealed samples. The dissipation is proportional to the derivative, so as the APD curves flatten out when in deep contact the dissipation returns to zero. These features are illustrated in Fig. 4b, which takes into account the relative width and height of the attractive regions and the relative values of sin(*φ*) and A/A0 in each case to produce heuristic curves.

In both annealed and non-annealed samples, the energy dissipation was negative when the tip was slightly above the substrate (not touching the substrate), implying that energy was being given to the tip. This is explained by the existence of attractive forces which pulled the tip in near the lower extent of its travel, but were reduced on the return trip, resulting in a net addition of energy to the oscillating tip. When the tip was in weak contact, energy was dissipated consistent with the model of forming and breaking capillaries with each transit. When in full contact, the energy dissipation was near zero which is expected when the interaction is dominated by the tip-sample repulsion, a conservative force^48^. For the non-annealed samples, the energy addition in the attractive regime should have been approximately 3 times higher than the annealed samples due to the 3 times higher phase increase in the attractive region. When water was present, there was a large variation in the range and strength of the forces between the tip and WS_2_ and the tip and SiO_2_. However, on annealed samples the variation in APD curves, and thus the variation between successive trials’ FD curves and dissipation coefficient was reduced. This was consistent with our observation that the variation in step height was reduced by an order of magnitude in the annealed samples.

**Figure 5:**
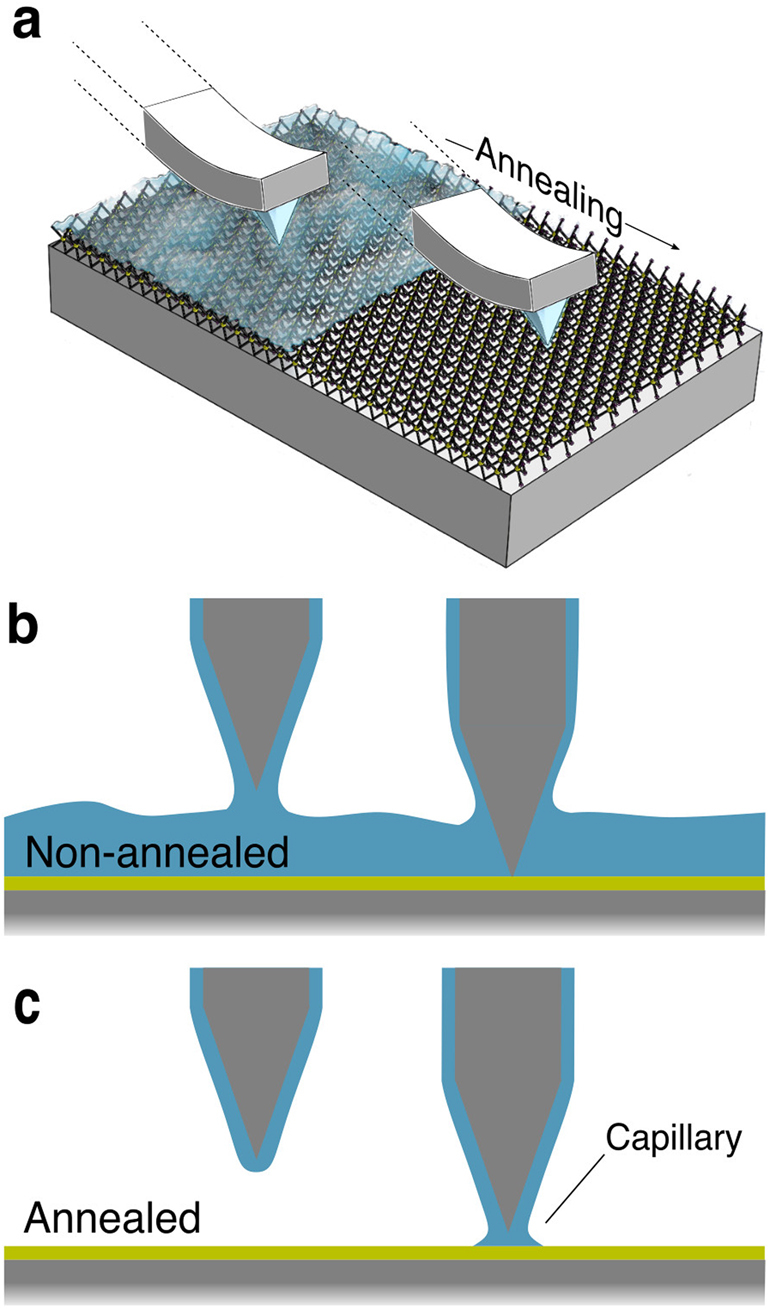
Illustration of varying tip-sample forces for TMDs before and after annealing. In A, a water film is illustrated coating the surface of the tip and sample, and after annealing only on the tip. B is a side view of the case before annealing: The water layers on the tip and sample interact to produce capillaries which result in long range attractive forces, up to 6 nm from the surface. When in full contact, the net force can still be positive until the strong repulsion makes the net force repulsive, after approaching several nm. In C, the annealed sample, the long range capillaries are absent but capillaries can still form within a couple of nm from the surface, resulting in attractive forces.

The variation between the measured height of WS_2_ on SiO_2_ and bare SiO_2_ in an AFM image can be explained by the variation in their hydrophilicities (Fig. 5). While SiO_2_ is hydrophilic, with contact angle (CA) of 10–50° depending on sample preparation with a predominantly polar component, TMD surfaces have been shown to be entirely dispersive, not polar, and weakly hydrophilic (CA 70–90°)^49^. Aging in air over a week increases the CA on CVD-grown WS_2_ from 70° to 83° due to carbon contamination^50^. It is known that water layers form on surfaces in low to high RH environments, with thicker layers forming in higher RH and on more hydrophilic surfaces. These water layers have been directly observed and quantified using force inversion from APD curves^51,52^. Capillary necks in AFM are a well-known phenomenon^53,54^. Capillaries have been directly observed in environmental SEM^55^. There was a clear difference in the nature of the tip-sample forces between WS_2_ and SiO_2_ when water was present, visible in the APD curves in Fig. 3, which accounts for the large variation in measured step height. When defining the set point, the perceived location of the sample surface is established, which results in incorrect measurement of step heights with varying tip-sample interactions. In amplitude feedback mode, the larger capillaries on the hydrophilic SiO_2_ result in stronger tip-sample interactions and greater amplitude reduction than on WS_2_. Thus, when water is present on the surface, the feedback loop approaches the tip closer on WS_2_ to reach the same set point, causing the WS_2_ to appear lower than the SiO_2_. To counteract this, the tip amplitude is increased and the set point lowered. This high tip amplitude results in lower relative amplitude reduction from the capillary forces, which we observed both in APD curves and in step measurements (Fig. 1a), in agreement with findings for other material systems^45^. The argument is similar for the phase feedback. Phase feedback only responds to phase dropping below 90°, and therefore, unlike amplitude mode, the tip approached through into the repulsive regime. With a greater width of the attractive region on WS_2_ in the PH curve, the TMD appears lower than the SiO_2_. This is remedied by increasing the amplitude and lowering the set point in order to reduce the relative magnitude of the capillary forces. This effect was observed when taking APD curves at varying amplitudes; the phase increases before contact (indicative of capillary forces) were reduced in height at higher amplitudes.

On annealed samples, the WS_2_ appeared inset for typical tip amplitudes and set points, which was corrected by using small amplitudes and larger set points, the opposite tool settings required when thick water layers were present. When a sample is annealed, there could still be water on the tip and water would reabsorb on the sample at a speed dependent on the surface’s hydrophilicity and the relative humidity^41^. Smaller capillaries can still form between the SiO_2_ and the tip on annealed samples, while having a harder time forming between the weakly hydrophilic WS_2_ and the tip. These strong tip-sample forces in the case of SiO_2_ cause the feedback loop not to approach as far on SiO_2_ to reach the same set point. This results in the SiO_2_ surface being measured higher than it is physically and the WS_2_ appearing inset. A low tip amplitude and high set point pushes the operation well into the repulsive regime, reducing the relative effect of capillaries and restoring the proper contrast between WS_2_ and SiO_2_. The step height variation was predominantly due to variations in hydrophilicity between samples. This can be corrected by using higher set points to operate further into the repulsive regime where capillary forces are not dominant. This effectively shifts on the PH curve to a region of lower variation between materials. Thus, different tool settings are required to prevent contrast inversion on non-annealed and annealed samples. When capillaries are present, high amplitudes and low set points reduce the effect of the capillaries on the tip, as seen in APH data as smaller angles in the attractive region at higher amplitudes. When capillaries are not present, low amplitudes and high set points ensure operation in the repulsive regime on both substrates, where repulsive forces dominate the tip-sample interactions.

The differences between annealed and non-annealed samples were more pronounced on WS_2_ than on SiO_2_, because WS_2_ is less hydrophilic than SiO_2_. Therefore, the WS_2_ does not re-wet as quickly in air after annealing. In addition, the bistability region was clearer on the annealed sample, shown on Fig. 3. The bistability results from a sudden transition from large net attractive force to a large net repulsive force, which we marked as an ad-hoc sample surface. The lack of a bistability on non-annealed samples was the result of total amplitude reduction within the attractive regime. The thick capillaries formed on non-annealed samples caused a net attractive force well into the region of tip-sample physical contact. On non-annealed WS_2_, the region of negative D was wider than on the non-annealed SiO_2_. This was a result of more rapid amplitude reduction in the attractive region on the WS_2_, (dA/dH > 1 causes D < 0), showing greater capillary effects on WS_2_ than SiO_2_ before annealing.

## Conclusion

We have shown that varying tip-surface interactions are the cause of step height measurement errors on TMDs, and contrast inversion in topographic images can be corrected by appropriate set point and tip amplitude: namely high amplitudes when capillaries are present and low amplitudes with high set points on annealed samples. We have found that water layers are the primary cause, with other forces resulting in errors an order of magnitude smaller. Due to the wettability difference between SiO_2_ and WS_2_, when water is present in the system, it is not possible to measure the step height with suitable accuracy (+/− 0.05 nm) without fully characterizing the tip-sample forces and finding the true sample surface. High vacuum annealing^32^ or scanning in liquid would alter the step height by removing potential contaminants, which would only allow one to find the step from pristine SiO_2_ to pristine WS_2_. Topographic measurements of TMD samples that have undergone processing (*i.e*., CVD growth or layer transfer) should include and characterize contaminants through APD and FD curves, because they will contain the contaminant layers. When water is present on the sample surface, high amplitudes and low set points correct contrast inversion due to reduced capillary forces, causing the WS_2_ to appear higher than the SiO_2_. When water has been removed from the sample surface, low amplitudes and high set points produce images with correct contrast. This is due to low amplitudes and high set points operating well into the repulsive regime, where the relative effect of capillaries is reduced. Exfoliated samples appeared higher than CVD grown samples, up to 8 nm, due to contaminant layers and unattached edges of the crystal lifted off the substrate. After high vacuum annealing to remove possible contaminants, the step heights were reduced to a similar range as CVD grown samples, including exhibiting contrast inversion, and the same analysis described above applies for CVD grown samples. dAFM’s sensitivity to surface chemistry and long range van der Waals forces, though occluding the topographic measurement, can be a boon to understanding the surface’s state in different environments. As a future work, we propose high sensitivity force volume measurements (taking APD curves in an array and performing force inversion) to elucidate the step height and contaminant layers in processed, real world TMD monolayers.

## Methods

### WS_2_ growth

WS_2_ monolayer samples were grown through chemical vapor deposition at 900 °C from solid sources (WO_3_ and S) on silicon substrates. They were confirmed to be monolayers through PL mapping. When not being measured, the WS_2_ samples were stored in Ar gas in an acrylic box with silica desiccant to prevent oxidation.

### AFM calibration

Samples were imaged in tapping mode using the Nanonics Imaging model MV-2000 AFM and in contact mode using the Nanonics Imaging model MV-1000. In order to convert the amplitude from volts to nanometers, the tip amplitude and piezo height was recorded in a separate tip approach and retract mapping on sapphire in contact mode. Sapphire was used because the hard surface would not deform during the tip’s contact with the surface. When in full contact the tip-amplitude response should be linear, assuming Hook’s law for the tip deflection. Then using a linear regression of the amplitude over the height, the slopes were found for the approach and retract curves, which agreed as expected. Taking the absolute value and the average of each slope, the conversion factor between volts and nanometers was found to be 0.008004 V/nm.

The conversion from piezo voltage to nm was obtained from imaging a standard sample, and was also assumed to be linear. The true tip-sample distance is obtained by shifting the height to be h = 0 at the sample surface, seen from the approach-retract curve, and adding the tip deflection, which is taken to be negative for downwards deflection. The tip’s spring constant was taken from the manufacturer’s estimate. Errors in spring constant only affect the magnitude of forces measured and do not affect the conclusions drawn from the features.

### APD curves

APD curves were taken using the Nanonics MV2000 and MV1000. In the data set presented in this paper, 10 trials were taken for each sample, interspersing tests on WS_2_ on SiO_2_ and bare SiO_2_, respectfully to prevent systematic errors due to tip degradation, before and after annealing. The APD experiments were performed with both low-Q silicon tips in a beam bounce setup and using a high-Q pulled fiber tip using piezo sensors to detect the tip amplitude and phase. Both experiments produced qualitatively similar results (only the high-Q results are shown in this work). Parameters were typically k = 3–9 N/m, Q = 1400, f = 33 kHz, and the in-air amplitude was A_0_ = 13 nm for the APD data shown. The tip was driven on resonance at constant frequency.

### Datasets

The datasets generated during and/or analyzed during the current study are available from the corresponding author on reasonable request.

## Electronic supplementary material

Supplementary Information

## References

[1] Peng B, Ang PK, Loh KP (2015). Two-dimensional dichalcogenides for light-harvesting applications. Nano Today.

[2] Eda G (2011). Photoluminescence from chemically exfoliated MoS_2_. Nano Lett..

[3] Mak KF (2010). Atomically thin MoS_2_: a new direct-gap semiconductor. Phys. Rev. Lett..

[4] Splendiani A (2010). Monolayers of W_x_Mo_1−x_S_2_ alloy heterostructure with in-plane composition variations. Nano Lett..

[5] Hong X (2014). Ultrafast charge transfer in atomically thin MoS_2_/WS_2_ heterostructures. Nat. Nanotechnol..

[6] Cai L (2015). Vacancy-induced ferromagnetism of MoS_2_ nanosheets. J. Am. Chem. Soc..

[7] Tongay S, Varnoosfaderani SS, Appleton BR, Wu JQ, Hebard AF (2012). Magnetic properties of MoS_2_: existence of ferromagnetism. Appl. Phys. Lett..

[8] Ataca C (2011). Mechanical and electronic properties of MoS2 nanoribbons and their defects. Phys. Chem., C.

[9] Shi W (2015). Superconductivity series in transition metal dichalcogenides by ionic gating. Sci. Rep..

[10] Jo S, Costanzo D, Berger H, Morpurgo AF (2015). Electrostatically induced superconductivity at the surface of WS_2_. Nano Lett..

[11] Hsu Y, Vaezi A, Fischer MH, Kim E (2017). Topological superconductivity in monolayer transition metal dichalcogenides. Nat. Commun..

[12] Su L, Yu Y, Cao L, Zhang Y (2017). *In situ* monitoring of the thermal-annealing effect in a monolayer of MoS_2_. Phys. Rev. Appl..

[13] Yu Y (2016). Engineering substrate interactions for high luminescence efficiency of transition-metal dichalcogenide monolayers. Adv. Funct. Mater..

[14] Yang R, Zheng X, Wang Z, Miller CJ, Feng PXL (2014). Multilayer MoS_2_ transistors enabled by a facile dry-transfer technique and thermal annealing. J. Vac. Sci. & Technol. B.

[15] Kang KN, Godin K, Yang E-H (2015). The growth scale and kinetics of WS_2_ monolayers under varying H_2_ concentration. Sci. Rep..

[16] Kang K (2017). Graphene-assisted antioxidation of tungsten disulfide monolayers: substrate and electric-field effect. Adv. Mater..

[17] Hong, J. W., Sang-il Park, and Z. G. Khim. Measurement of hardness, surface potential, and charge distribution with dynamic contact mode electrostatic force microscope. *Review of Scientific Instruments* **70**, 3, 1735–1739 (1999).

[18] Butt H-J (1991). Measuring electrostatic, van der Waals, and hydration forces in electrolyte solutions with an atomic force microscope. Biophysical Journal.

[19] Gotsmann B (1999). Conservative and dissipative tip-sample interaction forces probed with dynamic AFM. Physical Review B.

[20] Saenz JJ (1987). Observation of magnetic forces by the atomic force microscope. Journal of Applied Physics.

[21] Zitzler L, Herminghaus S, Mugele F (2002). Capillary forces in tapping mode atomic force microscopy. Phys Rev B.

[22] Gutiérrez HR (2013). extraordinary room-temperature photoluminescence in triangular WS_2_ monolayers. Nano Lett..

[23] Cong C (2014). Synthesis and optical properties of large-area single-crystalline 2D semiconductor WS_2_ monolayer from chemical vapor deposition. Adv. Opt. Mater..

[24] Knoll R, Magerle K, Krausch G (2001). Tapping mode atomic force microscopy on polymers: where is the true sample surface?. Macromolecules.

[25] Verdaguer A (2012). Water-mediated height artifacts in dynamic atomic force microscopy. Phys. Chem. Chem. Phys..

[26] Palacios-Lidón E, Munuera C, Ocal C, Colchero J (2010). Contrast inversion in non-contact dynamic scanning force microscopy: what is high and what is low?. Ultramicroscopy.

[27] Chen, X., Zhang, L., Zhao, Y., Wang, X. & Ke, C. Graphene folding on flat substrates. *J. Appl. Phys*. 116 (2014).

[28] Solís-Fernández P (2010). Determining the thickness of chemically modified graphenes by scanning probe microscopy. Carbon.

[29] Bar G, Thomann Y, Brandsch R, Cantow H-J, Whangbo M-H (1997). Factors affecting the height and phase images in tapping mode atomic force microscopy: study of phase-separated polymer blends of poly(ethene- co -styrene) and poly(2,6-dimethyl-1,4-phenylene oxide). Langmuir.

[30] Brandsch R, Bar G, Whangbo M-H (1997). On the factors affecting the contrast of height and phase images in tapping mode atomic force microscopy. Langmuir.

[31] Ebenstein Y, Nahum E, Banin U (2002). Tapping mode atomic force microscopy for nanoparticle sizing: tip-sample interaction effects. Nanoletters.

[32] Neves BRa, Leonard DN, Salmon ME, Russell PE, Troughton EB (1999). Observation of topography inversion in atomic force microscopy of self-assembled monolayers. Nanotechnology.

[33] Santos S, Barcons V, Christenson HK, Font J, Thomson NH (2011). The intrinsic resolution limit in the atomic force microscope: implications for heights of nano-scale features. PLoS One.

[34] Mechler Á (2005). Anomalies in nanostructure size measurements by AFM. Phys. Rev. B.

[35] Nemes-Incze P, Osváth Z, Kamarás K, Biró LP (2008). Anomalies in thickness measurements of graphene and few layer graphite crystals by tapping mode atomic force microscopy. Carbon.

[36] Shearer CJ, Slattery AD, Stapleton AJ, Shapter JG, Gibson CT (2016). Accurate thickness measurement of graphene. Nanotechnology.

[37] Xu K, Cao P, Heath JR (2010). Graphene visualizes the first water adlayers on mica at ambient conditions. Science.

[38] Dollekamp E, Bampoulis P, Poelsema B, Zandvliet HJW, Kooij ES (2016). Electrochemically induced nanobubbles between graphene and mica. Langmuir.

[39] Kuhle A, Sorensen AH, Bohr J (1997). Role of attractive forces in tapping tip force microscopy. J. Appl. Phys..

[40] Horcas. I (2007). WSXM: A software for scanning probe microscopy and a tool for nanotechnology. Rev. Sci. Instrum..

[41] Amadei CA, Tang TC, Chiesa M, Santos S (2013). The aging of a surface and the evolution of conservative and dissipative nanoscale interactions. J. Chem. Phys..

[42] Chen X (1998). Interpretation of tapping mode atomic force microscopy data using amplitude-phase-distance measurements. Ultramicroscopy.

[43] Payam AF, Martin-Jimenez D, García R (2015). Force reconstruction from tapping mode force microscopy experiments. Nanotechnology.

[44] García R, San Paulo A (1999). Attractive and repulsive tip-sample interaction regimes in tapping-mode atomic force microscopy. Phys. Rev. B.

[45] Santos S, Verdaguer A, Chiesa M (2012). The effects of adsorbed water layers on the apparent height of nanostructures in ambient amplitude modulation atomic force microscopy. J. Chem. Phys..

[46] Cleveland JP, Anczykowski B, Schmid AE, Elings VB (1998). Energy dissipation in tapping-mode atomic force microscopy. Appl. Phys. Lett..

[47] Tamayo J, García R (1998). Relationship between phase shift and energy dissipation in tapping-mode scanning force microscopy. Appl. Phys. Lett..

[48] Paulo ÁS, García R (2001). Tip-surface forces, amplitude, and energy dissipation in amplitude-modulation (tapping mode) force microscopy. Phys. Rev. B.

[49] Annamalai M (2016). Surface energy and wettability of van der Waals structures. Nanoscale.

[50] Chow PK (2015). Wetting of mono and few-layered WS_2_ and MoS_2_ films supported on Si/SiO_2_ substrates. ACS Nano.

[51] Calò A, Domingo N, Santos S, Verdaguer A (2015). Revealing water films structure from force reconstruction in dynamic AFM. J. Phys. Chem. C.

[52] Fukuma T, Ueda Y, Yoshioka S, Asakawa H (2010). Atomic-scale distribution of water molecules at the mica-water interface visualized by three-dimensional scanning force microscopy. Phys. Rev. Lett..

[53] García R, Calleja M, Pérez-Murano F (1998). Local oxidation of silicon surfaces by dynamic force microscopy: nanofabrication and water bridge formation. Appl. Phys. Lett..

[54] Kumar K (2011). A study on carbon-nanotube local oxidation lithography using an atomic force microscope. IEEE Trans. Nanotechnol..

[55] Weeks BL, Vaughn MW, De Yoreo JJ (2005). Direct imaging of meniscus formation in atomic force microscopy using environmental scanning electron microscopy. Langmuir.

