# Supplementary material for: Reduction in Step Height Variation and Correcting Contrast Inversion in Dynamic AFM of WS2 Monolayers
Source: Sci Rep. 2017 Dec 19;7:17798. doi: 10.1038/s41598-017-18077-4 (PMC5736643; doi:10.1038/s41598-017-18077-4)
Supplement: Supplementary file 1 — Supplementary Information [file 41598_2017_18077_MOESM1_ESM.pdf]

Reduction in Step Height Variation and Correcting Contrast Inversion in Dynamic AFM of WS_2 Monolayers

Supplementary Figures

Kyle Godin, Christian Cupo, and Eui-Hyeok Yang

Department of Mechanical Engineering, Stevens Institute of Technology, Hoboken, New Jersey 07030, United States

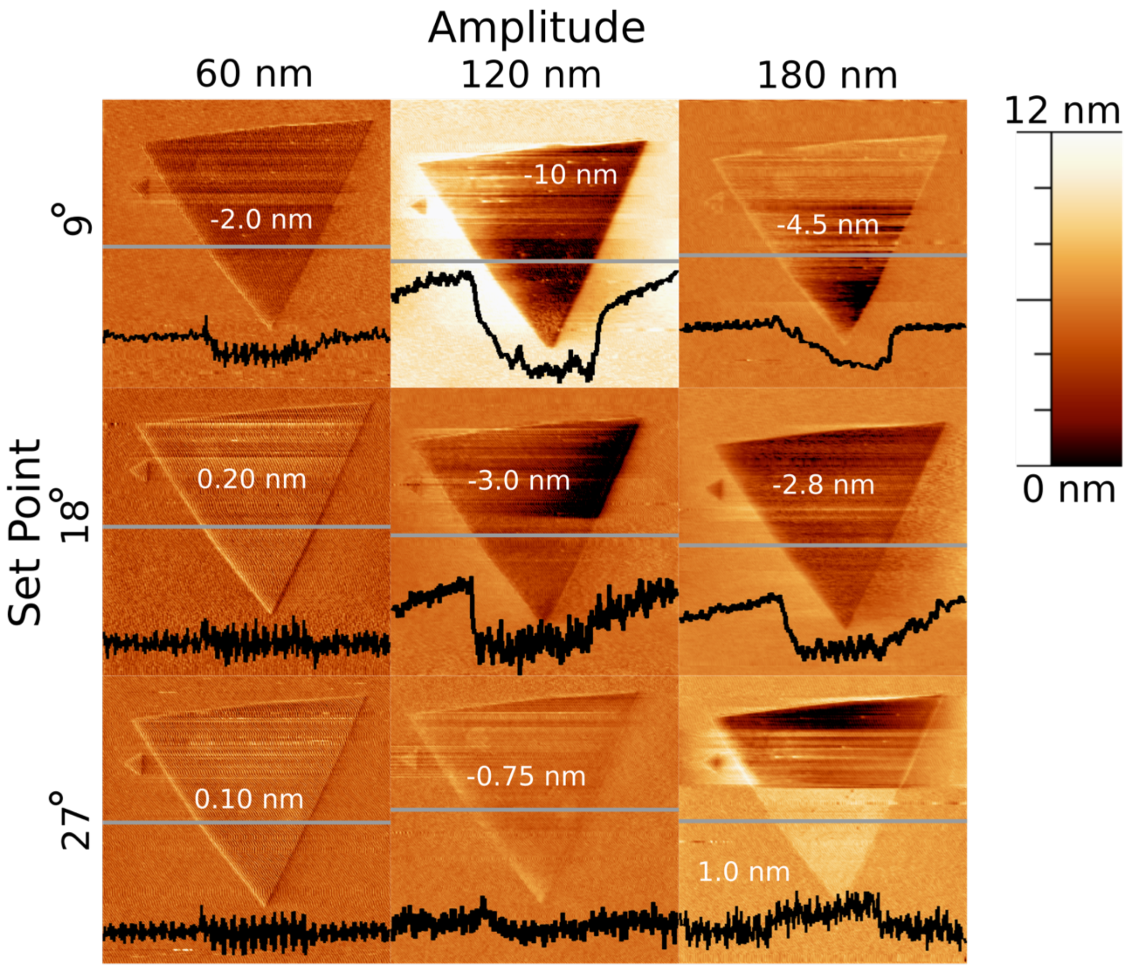

Figure S1: AFM topographic images of annealed CVD WS_2 monolayers on SiO_2. The WS_2 appears inset into the substrate except at low amplitudes and high set points. The bottom right image (180 nm, 27°) shows a switching of contrast mid-scan, attributed to operation near the bistability. This is in contrast to the results for the same experiment performed on as-grown samples shown in Figure 1a, where high amplitudes produce correct contrast.
